# Comparison of the effects of hydroxychloroquine and corticosteroid treatment on proteinuria in IgA nephropathy: a case-control study

**DOI:** 10.1186/s12882-019-1488-6

**Published:** 2019-08-05

**Authors:** Ya-zi Yang, Pei Chen, Li-Jun Liu, Qing-Qing Cai, Su-Fang Shi, Yu-Qing Chen, Ji-Cheng Lv, Hong Zhang

**Affiliations:** Renal Division, Key Laboratory of Renal Disease, Ministry of Health of China, Peking University First Hospital, Institute of Nephrology, Peking University, Beijing, 100034 People’s Republic of China

**Keywords:** IgA nephropathy, Hydroxychloroquine, Corticosteroid, Proteinuria

## Abstract

**Background:**

Hydroxychloroquine (HCQ), a well-known immunomodulator, has recently been found to be a promising and safe anti-proteinuric agent for treating IgA nephropathy (IgAN). We aimed to compare the efficacy and safety of HCQ and corticosteroid treatment in patients with IgAN.

**Methods:**

This is a case-control study. Ninety-two patients with IgAN who received HCQ in addition to routine renin-angiotensin-aldosterone system inhibitors (RAASi) therapy were included. Ninety-two matched historical controls who received corticosteroids were selected by propensity score matching. The clinical data over 6 months were compared.

**Results:**

Baseline proteinuria levels were comparable between the HCQ and corticosteroid groups (1.7 [1.2, 2.3] vs. 1.8 [1.3, 2.5] g/d, *p* = 0.96). The percentage reduction in proteinuria at 6 months was smaller in the HCQ group than in the corticosteroid group (− 48.5% [− 62.6, − 31.4] vs. -62.9% [− 81.1, − 34.9], *p* = 0.006). The time averaged proteinuria within the 6 months of observation was comparable for the HCQ and corticosteroid groups (1.1 [0.8, 1.5] vs. 1.1 [0.5, 1.8] g/d, *p* = 0.48). The cumulative frequency of patients with a 50% reduction in proteinuria during the study was also comparable between the two groups (52.2% vs. 62.0%, *p* = 0.25). However, six of the 92 (6.5%) patients suffered from severe adverse events (SAEs) in the corticosteroid group, while no SAEs were observed in the HCQ group (6.5% vs. 0%, *p* = 0.03).

**Conclusions:**

The antiproteinuric effect of HCQ might be slightly inferior to that of corticosteroids over 6 months in patients with IgAN who were deemed to be candidates for HCQ and not corticosteroids treatment. However, HCQ treatment was safer than corticosteroid treatment.

## Background

IgA nephropathy (IgAN) is the most prevalent type of primary glomerulonephritis worldwide [1]. Up to 30% of these cases will eventually progress to end-stage renal disease (ESRD) [2]. Most affected individuals develop chronic, slow-progressing renal injury. Lower proteinuria levels are generally accepted to be associated with slower renal function decline and lower ESRD risk in patients with IgAN [3]. Current treatment strategies primarily involve blood pressure control, renin-angiotensin-aldosterone system inhibitors (RAASi), and corticosteroids, which are recommended when the supporting therapies fail to reduce proteinuria levels to below 1 g/d [4]. Forty-five percent of patients achieve complete or partial proteinuria remission at 6 months with corticosteroid treatment [5]. However, up to 12–14% of corticosteroid users suffer severe adverse events (SAEs), including fatal severe infections [5, 6]. In addition, whether corticosteroids and immunosuppressive agents prevent renal failure remains controversial [7]. Thus, finding other treatment options is important.

Hydroxychloroquine (HCQ), which is a well-known immunomodulator that is widely used to treat autoimmunologic or inflammatory diseases, has been recently found to be a promising and safe antiproteinuric agent for treating IgAN. HCQ, in addition to routine RAASi treatment, effectively reduced proteinuria and improved the proteinuria remission frequency within 6 months [8, 9]. In our previous cohort study, HCQ in addition to an RAASi reduced proteinuria by − 43% [− 57, − 12], which was greater than the percentage change in proteinuria in the RAASi-only group (− 19% [− 46, 17], *p* = 0.01, [8]. The efficacy of HCQ in addition to optimized RAASi in patients with IgAN was further confirmed in randomized controlled clinical trial (NCT02942381, AJKD, in press). We found that the percentage change in proteinuria at 6 months was significantly different between the HCQ group and the placebo group (− 48.4% [− 64.2, − 30.5] vs. 10.0% [− 38.7, 30.6%], *p* < 0.001). However, no studies have compared the antiproteinuric effects of HCQ and a corticosteroid in IgAN.

In this study, we compared the efficacy and safety of HCQ and a corticosteroid in IgAN patients with persistent proteinuria.

## Methods

### Study population

We used a case-control design and adhered to STROBE guidelines/methodology. We reviewed the medical records from an IgAN database at Peking University First Hospital. This database contained 1363 patients biopsied from 1994 to 2018. Patients with IgAN who received HCQ treatment were included in this study. The exclusion criteria included the use of corticosteroids or immunosuppressive agents within the three previous months, RAASi treatment that was insufficient to ensure the patient was receiving the maximum labeled or tolerated dose according to KDIGO guidelines for IgAN within at least the three previous months, concomitant connective tissue disease, pregnancy or lactation, or macular degeneration. For each patient who received HCQ treatment, we selected one matched control who received corticosteroid therapy by performing propensity score matching based on age, sex, initial proteinuria level, estimated glomerular filtration rate (eGFR) and mean arterial pressure (MAP) level. We selected the control with the closest propensity score (within 0.2 SD) to each HCQ user in a 1:1 fashion and discarded the HCQ users without a suitable match and the remaining controls. The number of matching pairs determined the sample size. Patients with crescentic IgAN (defined by crescents in more than 50% of glomeruli), minimal renal disease changes with IgA deposits, acute or subacute tubulointerstitial nephritis, nephrotic syndrome (proteinuria level ≥ 3.5 g/d and serum albumin ≤30 g/L), more than a 30% decline in eGFR in the previous 6 months, acute kidney injury, and malignant hypertension were excluded in both groups. The study was approved by an independent ethics committee at the Peking University First Hospital.

### Interventions

In the HCQ group, the HCQ dose varied according to the baseline eGFR. The dose was 0.2 g twice daily for eGFRs greater than 45 ml/min/1.73 m^2^, and the dose was 0.1 g twice or thrice daily for eGFRs between 30 and 45 ml/min/1.73 m^2^; however, the dose was 0.1 g once daily for eGFRs between 15 and 30 ml/min/1.73 m^2^ [8]. Corticosteroid treatment was usually initiated when the patients presented with proteinuria > 1 g/d after supportive therapy for > 3 months. For patients with certain amounts of crescent or necrotizing lesions according to their renal biopsy, the treating physician may have added a corticosteroid immediately according to their discretion. Corticosteroid treatment included oral prednisone or prednisolone (0.8–1.0 mg/kg/d, maximum 60 mg/d) for 2 months. This treatment was tapered by 5 mg every 2 weeks and stopped within 6 to 8 months. Another corticosteroid regimen was bolus injections (i.v.) of methylprednisolone (500 mg) for 3 days at 1, 3 and 5 months, followed by prednisone (15 mg/d p.o.) for 6 months [6].

The patients were observed for 6 months or until the termination of HCQ/corticosteroid treatment or the addition of corticosteroids/immunosuppressive agents in HCQ users. Urinary protein excretion and eGFRs calculated by the CKD-EPI formula using serum creatinine (Scr) at 2, 4, and 6 months were assessed.

### Outcome measures

The primary outcome was the percentage change in proteinuria from baseline to 6 months. The secondary outcomes included the percentage change in proteinuria from baseline to 2 and 4 months; the cumulative frequency of patients with a 50% decrease in proteinuria; the time-averaged proteinuria (TA proteinuria), which was calculated as the weighted mean of all the measurements during follow-up, with the weight representing the time elapsed since the previous measurement [5, 8]; and the eGFR during follow-up.

SAEs were defined as any untoward medical occurrence that met one or more of the following criteria: resulted in death, was life-threatening, required inpatient hospitalization or prolongation of existing hospitalization, resulted in persistent or significant disability, severe infection that required hospitalization, osteonecrosis or bone fracture, gastrointestinal hemorrhage or perforation, new-onset diabetes mellitus (DM), new-onset cataract or fundus lesions, severe liver dysfunction or allergies that required hospitalization, and major cardio-cerebral vascular disease (including fatal/nonfatal myocardial infarction, fatal/nonfatal stroke, and heart failure) [6]. Adverse events (AEs) were collected according to the medical records. Patients receiving HCQ treatment were referred to ophthalmologist for retinal evaluation every 3–6 months.

### Statistical analysis

Normally distributed data are presented as the mean ± SD, and non-normally distributed data are presented as medians (Q25, Q75). The categorical data are summarized as counts and percentages. The baseline characteristics of the two groups were compared using the independent samples t test, Wilcoxon signed-rank test (for continuous variables) or χ^2^ test (for nominal variables) as appropriate. The cumulative frequency of patients with a 50% decrease in proteinuria was estimated using the Kaplan-Meier method, and time represented the period from baseline to the first occurrence of a 50% decrease in proteinuria.

All missing information was treated as missing data without imputation. Sensitivity analyses in which the missing observations were filled by carrying the last observation forward were performed, as well as an analysis using the subset of patients who received corticosteroid treatment after routine sufficient RAASi treatment for at least 3 months.

*P*-values less than 0.05 were considered statistically significant. Statistical analyses were performed using Stata 14.0 (Stata Corp., College Station, TX, USA) and SPSS 19.0 (SPSS Inc., Chicago, IL, USA).

## Results

### Baseline characteristics

**Fig. 1:**
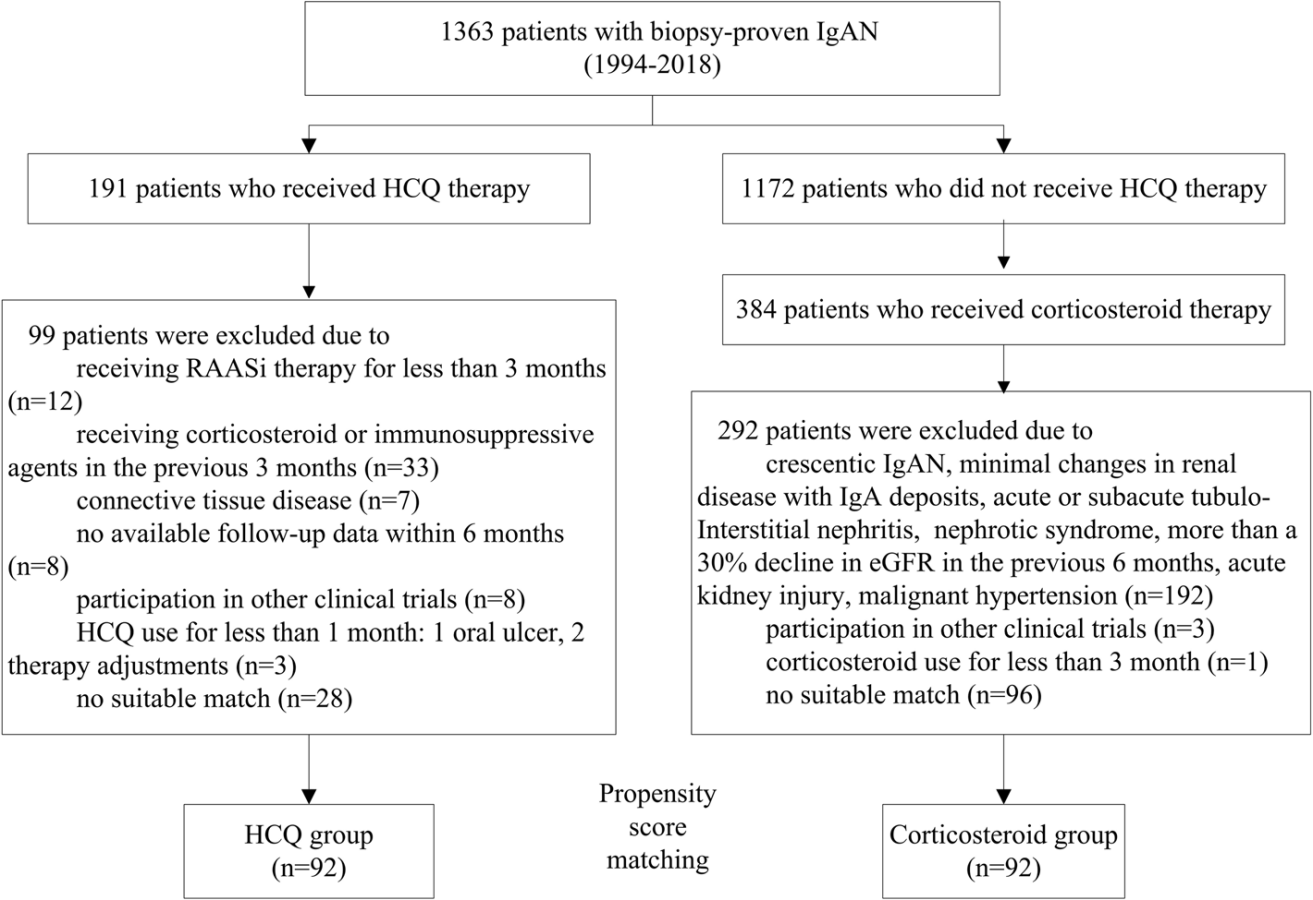
Study recruitment/inclusion flowchart

In total, 92 of 191 patients with IgAN who received HCQ treatment for 6.0 (4.1, 8.3) months from May 2013 to June 2018 were included in this study. Sixty-five of these HCQ patients overlapped with the 90 patients presented in our previous cohort study [8]. Ninety-two historical controls who received corticosteroid treatment and were matched for age, sex, initial proteinuria level, eGFR, and MAP level were selected by propensity score matching (Fig. 1). The baseline characteristics of the two groups are shown in Table 1. The baseline proteinuria levels (1.7 [1.2, 2.3] vs. 1.8 [1.3, 2.5] g/d, *p* = 0.96) and eGFRs (56.8 ± 20.4 vs. 55.2 ± 22.9 ml/min/1.73 m^2^, *p* = 0.61) were comparable between the HCQ and corticosteroid groups. Forty-two percent of the patients in the corticosteroid group received immunosuppressive agent treatment during the study.

**Table 1:** Baseline characteristics

	HCQ group (*n* = 92)	Corticosteroid group (n = 92)	*p* value
Age (years)	37.0 ± 10.0	37.2 ± 12.6	0.91
Sex (male/female)	46/46	46/46	1.00
MAP (mmHg)	90.6 ± 9.3	90.5 ± 9.2	0.96
Scr (μmol/L)	119.8 ± 37.7	127.6 ± 53.8	0.26
Baseline eGFR (ml/min/1.73 m^2^)	56.8 ± 20.4	55.2 ± 22.9	0.61
Baseline UTP (g/d)	1.7 (1.2, 2.3)	1.8 (1.3, 2.5)	0.96
Oxford classification*			
M 0/1	40/48	50/38	0.13
E 0/1	58/30	48/40	0.12
S 0/1	24/64	27/61	0.62
T 0/1/2	55/26/7	51/23/14	0.26
C 0/1/2	28/50/10	22/55/11	0.61
RAASi therapy (% of patients)	98.9	100	0.27
ACEI alone	38.0	48.9	
ARB alone	46.7	42.4	
ACEI plus ARB	14.1	8.7	
Immunosuppressive therapy (% of patients)	0	42.4	
Cyclophosphamide		32.6	
Mycophenolate mofetil		5.4	
Calcineurin inhibitors		2.2	
Leflunomide		1.1	
Tripterygium glycosides		1.1	
Aldosterone antagonist therapy (% of patients)	10.9	2.2	0.02
Traditional Chinese medicine (% of patients)	2.2	5.4	0.44

Abbreviations: *MAP* mean arterial pressure, *SCr* serum creatinine, *eGFR* estimated glomerular filtration rate (calculated using the CKD-EPI equation); *RAASi* renin-angiotensin-aldosterone system inhibitor, *ACEI* angiotensin-converting enzyme inhibitor, *ARB* angiotensin receptor blocker

*4 histological scores unavailable in the HCQ group because 2 patients received renal biopsy in other clinics, and the glomeruli were less than 8 on the renal specimen of the other 2 patients. 4 histological scores unavailable in the Corticosteroid group because 1 patient received renal biopsy in other clinic, 1 specimen was of poor quality that can’t be graded, and the glomeruli were less than 8 on the renal specimen of the other 2 patients

### Primary outcome

**Fig. 2:**
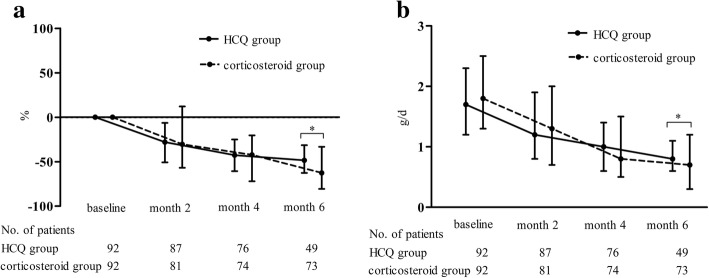
**a**-**b** Urinary protein excretion in the HCQ and corticosteroid groups during the follow-up period. The dots represent the median percentage change in proteinuria (**a**) or the proteinuria value (**b**); the bars represent the 25th and 75th percentiles. *p < 0.05; **p < 0.001

The percentage reduction in proteinuria from baseline to 6 months was smaller in the HCQ group than in the corticosteroid group (− 48.5% [− 62.6, − 31.4] vs. -62.9% [− 81.1, − 34.9], *p* = 0.006; Fig. 2a). The median proteinuria level at 6 months was higher in the HCQ group than in the corticosteroid group (0.8 [0.6, 1.1] vs. 0.7 [0.3, 1.1] g/d, *p* = 0.02; Fig. 2b). Additional analyses with the imputation of missing information confirmed significant differences between the two groups. However, in further sensitivity analyses, there were no significant differences in the percentage reduction in proteinuria or the proteinuria levels at 6 months between the HCQ group (*n* = 92) and the corticosteroid subset group (*n* = 42) that received routine sufficient RAASi treatment at least 3 months before corticosteroid treatment (− 48.5% [− 62.6, − 31.4] vs. -58.8% [− 73.2, − 26.5], *p* = 0.17; 0.8 [0.6, 1.1] vs. 0.7 [0.5, 1.6] g/d, *p* = 0.95).

### Secondary outcomes

**Fig. 3:**
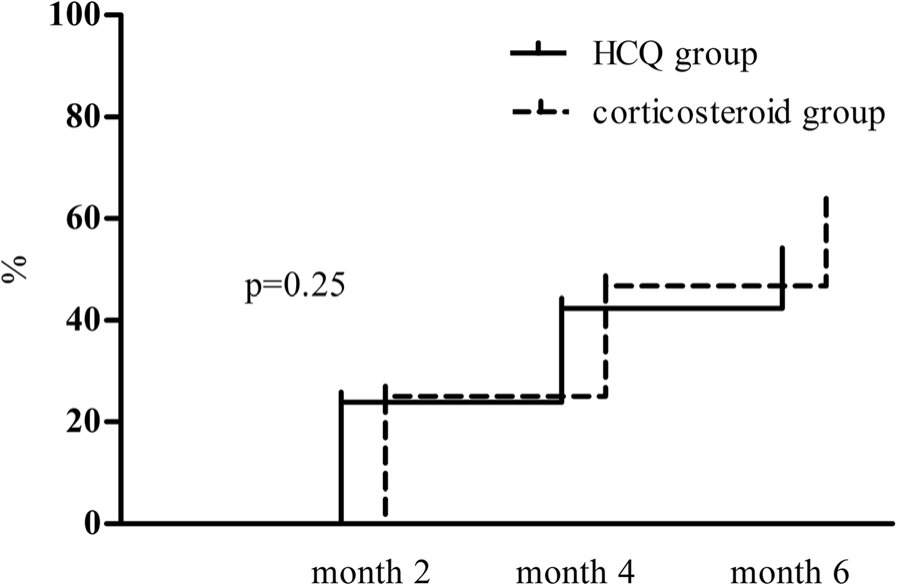
Cumulative frequency of a 50% reduction in proteinuria during the follow-up period

**Fig. 4:**
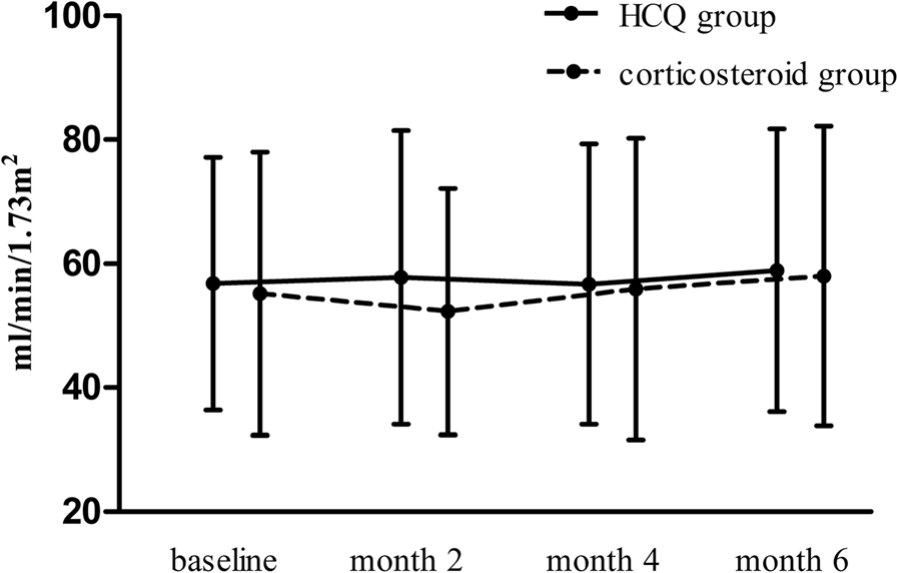
eGFRs in the HCQ and corticosteroid groups during the follow-up period. The dots represent the mean eGFR; the bars represent the standard deviation. * *p* < 0.05; ** *p* < 0.001

There were no statistically significant differences in the changes in proteinuria reductions from baseline to 2 and 4 months between the HCQ group and the corticosteroid group (at 2 months: − 28.0% [− 50.8, − 6.4] vs. -29.8% [− 56.8, 12.9], p = 0.95; at 4 months: − 42.6% [− 60.6, − 25.0] vs. -42.3% [− 72.1, − 20.4], *p* = 0.40). The cumulative frequency of patients with a 50% decrease in proteinuria within 6 months was 52.2% in the HCQ group, which was comparable with that in the corticosteroid group (62.0%, *p* = 0.25, Fig. 3). No significant difference was found in the TA proteinuria values between the HCQ group and the corticosteroid group (1.1 [0.8, 1.5] vs. 1.1 [0.5, 1.8] g/d, *p* = 0.48). There was also no significant difference in eGFRs at 6 months (Fig. 4).

### Safety and AEs

Six of 92 (6.5%) patients suffered SAEs in the corticosteroid group, while no SAEs were observed in the HCQ group (6.5% vs. 0%, *p* = 0.03). The SAEs included 1 death (due to pneumocystis pneumonia), severe infections necessitating hospitalization (*n* = 3), gastrointestinal bleeding (*n* = 1), DM (*n* = 1) and hyperosmolar hyperglycemia (*n* = 1) in the corticosteroid group. In the corticosteroid group, no statistically significant difference in the frequency of SAEs was found between the patients receiving corticosteroid monotherapy or with other immunosuppressants as well (3/53 vs. 3/39, *p* = 0.70). More AEs were observed in the corticosteroid group than in the HCQ group (34/92 vs. 9/92, *p* < 0.001). One patient in the HCQ group presented with an eGFR reduction of 29.0% from baseline to 4 months, and one patient presented in the HCQ group with an eGFR reduction of 33.4% at 6 months. Both of these reductions resulted in therapy adjustments in the HCQ group. None of the AEs in the HCQ group led to hospitalization. The AEs in both groups are listed in Table 2.

**Table 2:** SAEs and AEs in the HCQ and corticosteroid groups

	HCQ group, n = 92	Corticosteroid group, n = 92	*p* value^e^
SAEs			0.03
No. of events			
0	92	86	
1	0	5	
≥ 2	0	1	
Event details			
Death^a^		1	
Severe infection		3	
Pneumonia^a^		2	
Gastrointestinal infection		1	
Gastrointestinal bleeding^a^		1	
New-onset diabetes mellitus		1	
Hyperosmolar hyperglycemia		1	
AEs			< 0.001
No. of events			
0	81	52	
1	6	19	
≥ 2	3	15	
Event details			
Cardiovascular effects			
Palpitations^b^	1	4	
Exertional dyspnea	0	1	
Gastrointestinal effects			
Liver dysfunction	1	5	
Nausea^b^	1	1	
Abdominal discomfort/pain	0	5	
Bloating	0	3	
Constipation	0	1	
Bone and muscle effects			
Arthralgia	0	8	
Arthrocele	0	1	
Myalgia	0	2	
Muscle fasciculation	0	2	
Muscle enzyme elevation	0	1	
Numbness	0	1	
Hand tremors	0	1	
Ostealgia	0	1	
Neuropsychiatric effects			
Dizziness	0	1	
Insomnia	0	4	
Ophthalmologic effects			
Intraocular pressure elevation	1	0	
Blurred vision	0	2	
Ocular swelling pain	0	1	
Urogenital effects			
eGFR reduction	2	0	
Menstrual disorder	0	2	
Mucocutaneous effects			
Pruritus^c^	2	0	
Skin pigmentation^b^	1	0	
Ecchymosis	0	1	
Oral ulcer	0	1	
Acne	0	1	
Desquamation	1	0	
Papules	0	3	
Folliculitis	0	1	
Alopecia^c^	1	1	
Hematologic effects			
Lymphocytopenia	0	1	
Anaphylactic effects			
Dyspnea^d^	1	0	
Rashes^d^	1	0	

Abbreviations: *SAEs* serious adverse events, *AEs* adverse events, *eGFR* estimated glomerular filtration rate

^a, b, c, d^These AEs occurred in the same patient

^e^Comparison of the proportion of patients with at least 1 event using Fisher’s exact test

## Discussion

In the current study, we compared the percentage change in proteinuria in IgAN patients who received HCQ with matched historical controls who received corticosteroid therapy. The percentage change in proteinuria over 6 months was slightly lower in the HCQ group than in the corticosteroid group. The cumulative frequency of a 50% reduction in proteinuria and TA proteinuria was comparable between the two groups. However, the risk of SAEs and AEs was notably higher in the corticosteroid group than in the HCQ group. Thus, this study suggests that the antiproteinuric effect of HCQ might be slightly inferior to that of corticosteroids over 6 months in IgAN patients who were deemed to be candidates for HCQ and not corticosteroids treatment, but HCQ treatment was safer than corticosteroid treatment.

Although the pathogenesis of IgAN is not yet fully understood, an immune complex containing galactose-deficient IgA1 (Gd-IgA1) that mediates inflammation is thought to be vital in the process of renal injury [10]. Currently, immunosuppressive therapy, mainly corticosteroids, is recommended for patients with persistent proteinuria despite optimized RAASi treatment [4]. The risk of SAEs, including severe infections and incapacitating bone and endocrine disorders, is high, but the antiproteinuric efficacy of corticosteroids has undoubtedly been confirmed [5, 7]. Therefore, the investigation of new treatment strategies is necessary.

Recently, it was found that a 6-month regimen of HCQ in addition to routine supportive treatment improved the probability of partial proteinuria remission and reduced proteinuria with few SAEs [8, 9]. Early decline in proteinuria is associated with a lower risk for long-term renal outcomes in studies of IgAN (HR, per 50% reduction in proteinuria, 0.40; 95% CI, 0.32 to 0.48; *P* < 0.001, [11]. In this study, we chose the percentage reduction of proteinuria at 6 months as a surrogate end point, and found that the median proteinuria decreased by 48.5% at 6 months in HCQ group, which was consistent with the results of our previous study [8]. The percentage reductions were comparable between the HCQ and corticosteroid groups at 2 and 4 months, but the percentage reduction was lower in the HCQ group than in the corticosteroid group at 6 months. It seemed that HCQ was not as powerful as the corticosteroids at reducing proteinuria. However, 42% of patients in the corticosteroid group received immunosuppressive agent treatment during the study. In addition, because some of the patients received corticosteroids without sufficient RAASi therapy (due to the decisions of their physicians when certain amounts of crescent or necrotizing lesions were detected by biopsy), the median duration of RAASi therapy was less than 3 months in the corticosteroid group. Therefore, the anti-proteinuria effects of corticosteroids may have been overestimated in this study. Furthermore, in an additional sensitivity analysis of patients with routine sufficient RAASi therapy, there was no significant difference in the percentage change in proteinuria between the two groups. However the lack of statistical difference might be due to loss of power from comparing smaller subset, as the magnitude of difference was similar to the whole corticosteroid group (− 58.8% vs. -62.9%).

The immunomodulatory action of HCQ may result from its ability to affect lysosome stability and suppress antigen presentation, lymphocyte activation, cytokine synthesis and toll-like receptors (TLR) stimulation [12–14]. HCQ treatment led to a lower incidence of chronic kidney disease in systemic lupus erythmaetosus and rheumatoid arthritis patients, possibly due to reduced intra-renal inflammation [15, 16]. In our study, no difference in renal function during the 6 months of follow-up was observed between the HCQ group and the corticosteroid group. The reason for this might be that the study period was not long enough to draw a definitive conclusion about the different effects of the two treatment strategies on renal function. Most SAEs occurred in the first 3 months of corticosteroid treatment [5], and about half of the SAEs were recorded in the first 6 months of follow-up [6]. The frequency of SAEs in the corticosteroid group was 6.5%, including one death that resulted from severe infection; this frequency was consistent with those found in previous studies [6]. Moreover, we collected the AEs according to the medical records of the IgAN patients and found that as many as 37.0% of the patients receiving corticosteroid treatment complained that the side effects disturbed their daily life. These side effects included arthralgia, palpitations and insomnia. Though the effects were not lethal or disabling, the effects were strong enough to decrease the patients’ quality of life, a factor that has not received enough attention in IgAN patients. In contrast, the safety of HCQ has been well-documented in its history of use in rheumatology. HCQ was well tolerated in patients with IgAN. In addition, the rate of AEs was significantly lower in the HCQ group than in the corticosteroid group.

The major limitation of this study is that it was a retrospective study with a short follow-up period. As a retrospective study, the selection bias between patients chosen to receive steroids with or without immunosuppression and those who did not receive these drugs is inevitable. The era of diagnosis was earlier in the corticosteroid group than the HCQ group, because the HCQ treatment was introduced to IgAN patients only in recent years. As we excluded patients using corticosteroids or immunosuppressive agents within 3 months prior to HCQ use, it tended to exclude more severe or treatment resistant disease from the HCQ arm which might potentially favor the HCQ group. Since this is a retrospective and propensity matched study, the matched patients receiving corticosteroids will not represent the full spectrum of IgAN patients treated with corticosteroids. Moreover, no a priori power calculation limits interpretation of negative results. We did not view HCQ as an alternative treatment for IgAN patients with crescentic IgAN, minimal renal disease changes with IgA deposits, acute or subacute tubulointerstitial nephritis or nephrotic syndrome. In such cases, corticosteroids treatment still is the most suitable strategy for now. Since adverse events were collected retrospectively, patients on corticosteroids might have been followed more closely and thus more events captured which might lead to a biased estimate. Thus, the long-term antiproteinuric efficacy and safety comparisons between HCQ and corticosteroid therapy remain unknown. Multi-center clinical studies and mechanism research are needed in the future.

## Conclusions

In conclusion, the antiproteinuria effect of HCQ might be slightly inferior to that of corticosteroids over 6 months in patients with IgAN who were deemed to be candidates for HCQ and not corticosteroids treatment. However, HCQ treatment was much safer than corticosteroid treatment.

## Data Availability

The datasets used and/or analysed during the current study are available from the corresponding author on reasonable request.

## Abbreviations

ACEI: Angiotensin-converting enzyme inhibitor
AEs: Adverse events
ARB: Angiotensin receptor blocker
DM: Diabetes mellitus
eGFR: estimated glomerular filtration rate
ESRD: End-stage renal disease
Gd-IgA1: Galactose-deficient IgA1
HCQ: Hydroxychloroquine
IgAN: IgA nephropathy
MAP: Mean arterial pressure
PCR: Protein creatinine ratio
RAASi: Renin-angiotensin-aldosterone system inhibitors
SAEs: Severe adverse events
SCr: Serum creatinine
TA proteinuria: time-averaged proteinuria
TLR: Toll-like receptors
